# “We hope to, but…”: Chinese frontliners' barriers in providing psychosocial care for cancer survivors

**DOI:** 10.1016/j.apjon.2025.100725

**Published:** 2025-05-19

**Authors:** Ziqi Peng, Xiaohui Su, Suet Lin Hung

**Affiliations:** a Department of Social Work, Hong Kong Baptist University, Hong Kong, SAR, China; b School of Sociology and Anthropology, Sun Yat-sen University, Guangzhou, China

**Keywords:** Psychosocial oncology, Frontline professionals, Qualitative research, Cancer survivorship, Chinese, Barriers to care

## Abstract

**Objective:**

In recent years, there has been an emerging trend of psychosocial oncology caring strategies within the Chinese context. However, many survivors undergoing different stages of the cancer journey continue to report significant life disruptions and unmet psychosocial care needs. While the crucial role frontline professionals play in providing this care, little is known about the critical barriers they encounter. This study aims to provide a comprehensive understanding of the barriers Chinese frontline professionals face in providing psychosocial oncology care.

**Methods:**

This study employed a qualitative design with semi-structured interviews. Twenty oncology nurses and social workers with at least one year of experience in relevant specializations were recruited. The interview data were audio-recorded, transcribed, and thematically analyzed to identify themes.

**Results:**

Three dominant themes and subthemes were identified: (1) Overburdened clinical work and inadequate training, (2) Emotional and social struggles faced by survivors and their families that hinder the employment of psychosocial care, (3) System failures: social, structural, and policy barriers.

**Conclusions:**

The findings shed light on the complexities and barriers among frontline professionals in providing psychosocial oncology care. A central paradox emerged between professionals' desire to offer comprehensive care and the systemic barriers encountered, including limited institutional support and resources, and low public awareness. This study highlighted the need for a context-specific psychosocial oncology framework for research and practice. Interdisciplinary collaboration should be made among research, policy, and social education, which aims to enrich public awareness of psychosocial oncology and provide financial and resource support for practices.

## Introduction

Cancer survivorship is a process that provides continual care from diagnosis and treatment to rehabilitation.^1^^,^^2^ While the term of cancer survivor has varied interpretations, in this study, it refers to individuals who have been diagnosed with cancer, are undergoing treatment, or are in recovery.^3^^,^^4^ Psychosocial care plays a significant role in addressing the issues associated with cancer treatment, which provides effective non-medical care to improve the outcomes of cancer survivors.^1^^,^^5^^,^^6^ Psychosocial oncology care addresses the disruptions cancer causes in survivors' physical, psychological, and socioeconomic well-being.^6^, ^7^, ^8^, ^9^, ^10^ Specifically, psychosocial oncology care may improve cancer survivors’ outcomes related to late effects (accomplished sexual dysfunction, infertility, body image dysfunction, fatigue and sleep issues and risk of cancer recurrence) of treatment,^8^^,^^10^ mental health symptoms (including general depressive and anxiety symptoms, post-traumatic stress symptoms, coping issues, somatization, and even suicidal ideation),^8^^,^^9^ and social reintegration, including relationships and personal achievement.^8^^,^^11^^,^^12^ To support these goals, psychosocial oncology professionals provide a range of services throughout cancer survivorship. They screen for psychological symptoms, socioeconomic challenges, and late effects of cancer treatment and provide interventions such as information on body management, psychotherapy, post-hospital follow-up care, and financial assistance.^1^^,^^2^^,^^6^^,^^8^ These services help cancer survivors and their families manage psychosocial outcomes in cancer treatment and the recovery journey.

With increasing cancer diagnoses in China and improvement in healthcare, millions of newly diagnosed cases have become cancer survivors in recent years. The 5-year cancer survival rate stands at 43.3%.^2^^,^^13^^,^^14^ This trend highlights a growing number of cancer survivors facing physical, psychological, and socioeconomic challenges related to long-term treatment and the post-treatment period.^2^^,^^15^, ^16^, ^17^, ^18^ Psychosocial oncology within the Chinese context adopts a holistic approach, aimed at improving care for physical and psychological distress, social functioning, economic difficulties, and spiritual concerns.^19^ This care is implemented under specific Chinese cultural norms, folk beliefs, and stigma.^16^^,^^20^ For instance, seeking psychosocial support is often stigmatized, as an indication that a person has a mental illness.^16^ Studies from the 2010s have documented significant cultural and institutional challenges to psychosocial care for Chinese cancer survivors, including multi-level stigma and a lack of intervention framework.^16^^,^^20^ Recently, studies explored emerging psychosocial oncology interventions for the Chinese contexts, including peer support, psychotherapy, psychoeducation, and photovoice.^2^^,^^18^^,^^19^^,^^21^, ^22^, ^23^, ^24^, ^25^ These approaches addressed survivors' late effects and psychological distress while promoting holistic care for cancer survivors. Although previous studies have demonstrated the benefits of psychosocial care for the different populations of Chinese cancer survivors, recent evidence indicates that such care remains insufficient within Chinese healthcare in cancer care.^13^^,^^26^, ^27^, ^28^ Limited access to psychosocial care and unmet care needs result in ongoing suffering and struggle with psychosocial issues among cancer survivors.^13^^,^^26^^,^^27^^,^^29^ While the psychosocial needs of cancer survivors are a global issue, Chinese cancer survivors continue to experience significantly high levels of psychosocial distress, including high prevalence of depression, anxiety, and suicide.^30^^,^^31^ These unmet needs primarily include access to better information for cancer survivorship, psychological support for coping with mental health symptoms, and follow-up care to addresses physical well-being and socioeconomic reintegration.^10^^,^^13^^,^^25^, ^26^, ^27^

Several barriers contribute to the underestimation and underdevelopment of psychosocial care for Chinese cancer survivors, including limited support, resources, and approaches.^4^^,^^13^^,^^19^^,^^27^^,^^32^ Specifically, Chinese psychosocial cancer care is inadequately integrated with medical treatment, offering only basic pain screening, limited counseling services, and minimal institutional support.^4^^,^^19^ A biomedical focus on cancer treatment and disparities in services distribution further restricts Chinese psychosocial oncology care.^27^^,^^30^ For example, holistic psychosocial oncology care only operates in top-tier hospitals in developed cities, such as Beijing.^33^ From a human resources perspective, Chinese nurses are responsible for nearly 70% of psychosocial oncology care delivery under routine clinical demands, and with limited training and institutional support.^30^^,^^32^^,^^34^ Meanwhile, oncology social work in mainland China remains underdeveloped, with little evidence in research studies and a shortage of professionals.^19^^,^^33^ The current number of oncology social workers is extremely inadequate given the high demands of psychosocial needs.^19^^,^^33^ These challenges reflect longstanding cultural and systemic barriers that continue to hinder Chinese psychosocial oncology care in contemporary Chinese society.^16^^,^^30^

Despite the crucial role and primary responsibility in providing care for cancer survivors, research on the experiences and encountered barriers faced by Chinese frontline professionals in providing psychosocial care remains severely limited.^30^^,^^32^^,^^34^ The absence of these frontline experiences hinders a deeper understanding and insights into addressing the unmet needs of cancer survivors.^32^ As Botti et al.^5^ emphasized, identifying frontline barriers in psychosocial oncology care provides significant insights for developing effective strategies and intervention frameworks. This study seeks to examine the current barriers that hinder frontline professionals from addressing the psychosocial needs of Chinese cancer survivors. By identifying these challenges, we aim to shed light on the complexities of providing psychosocial oncology care and to inform policy and practice development of psychosocial oncology care within the Chinese health care context.

## Methods

### Study design

This study employed a qualitative design, using semi-structured interviews to explore frontline professionals' experiences with the barriers and challenges in providing psychosocial care for Chinese cancer survivors.^35^

### Data collection

Participants were recruited from mainland China using purposive sampling. Eligible frontline professionals, including nurses and social workers, were selected and invited based on the following inclusion criteria: (1) currently working with Chinese cancer survivors for at least one year, (2) self-reported experience providing psychosocial care to this population, (3) willing to disclose their personal experience in this field. These inclusion criteria were designed to reflect the different duties of oncology nurses and social workers in psychosocial oncology care.^5^^,^^19^^,^^36^

Oncology nurses in China are responsible for psychosocial care in clinical settings, focusing on alleviating bodily symptoms-related psychological distress, referring survivors with severe mental health symptoms, and educating survivor and their families in psychosocial care.^16^^,^^19^^,^^32^ In contrast, oncology social workers' duties feature in forming peer support groups or events, conducting psychotherapy, and acquiring funding for survivors with low socioeconomic status.^16^^,^^19^ These two specializations are forming an interdisciplinary collaboration to address the psychosocial needs of Chinese cancer survivors.^19^

This purposive sampling framework ensured the recruitment of professionals with relevant experience and excluded those with insufficient experience or not involved in psychosocial care, thereby enhancing the credibility of interview data.^35^ We involved maximum variation strategies to engage and include professionals with different specializations, years of experience, and regions for data transferability.^5^ Recruited participants who are proficient in speaking Mandarin or Cantonese.

A total of 20 frontline professionals were recruited as participants in this study. Additionally, five potential participants (not included in the total number of participants) withdrew their participation after reviewing the interview questions, citing their unwillingness to disclose.

### Procedures

The first and second authors distributed the recruitment advertisement to the oncology professionals' network in mainland China, using online and on-site posters. Eligible participants who were interested and met the criteria were provided with an informed consent form, which they signed before participating. The interview followed a five-section interview protocol developed by the first author and informed by existing studies.^5^^,^^26^^,^^27^ This interview guide was pilot-tested with professionals to ensure question clarity and relevance. A sample question included “Please describe in detail any challenges or difficulties you encountered in your experience of providing psychosocial care for cancer survivors.”

All interviews were conducted online via Tencent meeting by the first and second authors in private and quiet settings to ensure confidentiality. Both interviewers had years of PhD-level training in qualitative studies and were supervised by the third author throughout the data collection process. Each interview ranged from 60 to 70 minutes, which encompassed a section of demographic characteristics survey and semi-structured interviews. All interviews were audio-recorded, transcribed verbatim, and returned to participants for review to ensure data trustworthiness.

### Data analysis

Thematic analysis was employed to analyze the interview data.^37^ The interview data were reviewed and preprocessed by the first and second authors and uploaded to MAXQDA (version 24.8.0) for analysis. MAXQDA is a robust qualitative analysis software that enables collaborative coding, analyzing, and visualizing qualitative data.^38^

The analysis followed a three-step procedure with trustworthiness strategies. First, the two investigators independently analyzed the transcripts, identifying initial codes. Second, investigator triangulation was involved during the coding organization and merging process. The first and second authors analyzed the data independently, and all authors collaboratively reviewed and compared the generalized themes, ensuring the consolidation of the coding framework. Third, cross-checking was performed to resolve disagreements until a consensus was reached. This process led to the synthesis of the final themes and subthemes. Data saturation was achieved by 16 interviews. Four additional interviews were conducted to ensure all relevant themes and perspectives are adequately represented. To ensure the quality of reporting, the COREQ (COnsolidated criteria for REporting Qualitative research) checklist was used.

Participant quotes were translated from Chinese to English by the first and second authors. To ensure accuracy and cultural validity, all translations were verified through back-translation and reviewed by a peer researcher from mainland China.

## Results

### Characteristics of participants

Table 1 presents the demographic characteristics among the 20 participants. Of these, 18 were female (90%), and 2 were male (10%), with aged from 24 to 44 years (Mean ​​= ​​32.6, SD ​​= ​​5.2). Most of the participants were nurses (*n* ​​= ​​16, 80%), then social workers (*n* ​​= ​​4, 20%). These participants mainly resided in Guangdong province, mainland China (*n* ​​= ​​17, 85%). Participants reported experience in psychosocial oncology care ranging from 1 to 20 years (Mean ​​= ​​8.4, SD ​​= ​​5.7), and most held an undergraduate degree (*n* ​​= ​​17, 85%). In terms of specialization, the majority of participants worked in oncology (*n* ​​= ​​9, 45%), followed by bone cancer (*n* ​​= ​​4, 20%), pediatric oncology (*n* ​​= ​​3, 15%), nasopharyngeal oncology and interventional oncology (*n* ​​= ​​2, 10%).

**Table 1:** Participants' demographic characteristics (*N* ​​= ​​20).

Characteristics	*n* (%) or Mean ​​± ​​SD (range)
**Sex**	
Male	2 (10)
Female	18 (90)
**Age (years)**	32.6 ​​± ​​5.2 (24–44)
**Years of experience in psychosocial oncology care**	8.4 ​​± ​​5.7 (1–20)
**Educational level**	
Diploma	2 (10)
Undergraduate	17 (85)
Postgraduate	1 (5)
**Occupation**	
Nurse	16 (80)
Social worker	4 (20)
**Specialization**	
Oncology	9 (45)
Bone cancer	4 (20)
Pediatric oncology	3 (15)
Nasopharyngeal oncology	2 (10)
Interventional oncology	2 (10)
**Regions (province)**	
Guangdong	16 (80)
Shanghai^a^	2 (10)
Beijing^a^	2 (10)

a Province-level municipality city in China.

### Themes

Thematic analysis of the 20 participants' interviews identified three overarching themes with associated subthemes. Table 2 presents an overview of the generated themes and subthemes. The abbrevitation of data quotation, N representing nurse and S representing social worker.

**Table 2:** Summary of themes.

Themes	Subthemes
1. Overburdened clinical work and inadequate training	1a) Dilemma on human resources: Emotions, burdens, and turnover in clinical work 1b) Limited skills, knowledge, and supervision for psychosocial care 1c) The fear of medical disputes and liability uncertainty
2. Emotional and social struggles faced by survivors and their families that hinder the employment of psychosocial care	2a) Communication difficulties 2b) Emotional instability, isolation, and denial of psychosocial care 2c) The dynamic between survivors and their families
3. System failures: Social, structural and policy barriers	3a) Insufficient policy and institutional support for psychosocial care 3b) Low public awareness of psychosocial care 3c) Financial burden and insurance gaps in psychosocial care 3d) Geographical barriers to accessing care

### Theme 1: overburdened clinical work and inadequate training

#### Subtheme 1.1: dilemma on human resources: emotions, burdens, and turnover in clinical work

When interviewing participants about their experience with barriers to providing psychosocial care for Chinese cancer survivors, the most frequently expressed concerns were about their emotional struggles, perceived burdens, and high turnover due to the heavy workload. Specifically, these barriers encompassed a shortage of specialists trained in psychosocial care, increased stress and depressive mood relevant to handling additional psychosocial care beyond a heavy workload, and turnover related to emotional distress. These findings reflect the burden on frontline professionals by high patient volumes and limited resources, which can lead to high pressure in a clinical setting.

S1 Actually, it's really difficult to find such services in mainland China, I mean, they are relatively rare. So, we really hope that other organizations can step in and provide supplementary social workers to care for and support cancer survivors.

N11 The working hours… I mean, it's impossible for you to spend a full 20–30 minutes with a single patient in a clinical setting, providing them with psychosocial counseling.

N16 Today, I worked an extra hour and a half just to complete my treatment-related tasks. If I still have to do this (psychosocial care) and conduct ongoing assessments and follow-up on post-discharge psychosocial health—how much manpower would that require?

N7 The social workers in our department have provided a lot of help with our work. However, each social worker doesn't stay in the department for long, likely because of the psychological pressure they faced.

S4 Once I take off this uniform, I can't think about it anymore (the struggles of work)… If I keep dwelling on it, I'll feel very distressed, and it might even haunt me in the middle of the night.

#### Subtheme 1.2: limited skills, knowledge, and supervision for psychosocial care

Although frontline professionals are working under high pressure to meet both clinical and psychosocial care needs of cancer survivors, they remain committed to providing psychosocial care that they are able to offer. However, their efforts are constrained by limited skill, insufficient knowledge, and a lack of supervision. It hinders professionals' ability to deliver effective care to cancer survivors. These barriers reflect that these frontline professionals only rely on limited and unsystematic knowledge to address the unmet needs of Chinese cancer survivors. For example, a professional with limited communication skills in giving psychosocial care advice to a survivor. Additionally, they rarely received supervision to guide or assess their psychosocial care practices. The inadequate training and support contributed to feelings of stress and helplessness among professionals as they attempted to help cancer survivors.

N14 Actually, we haven't received any professional training in psychosocial care. If there were systematic training, it would be a win–win situation … there's no one giving me supervision on this work. Then, I have to rely on myself to figure things out, which takes a lot of time and becomes impossible to manage.

N9 We shouldn't casually give them any advice. Since we're not professionals, what if an unintentional action of ours ends up upsetting them?

N16 There is no systematic training. Sometimes, case sharing is performed, such as doing an emotional assessment … If psychosocial consultations can't resolve complex problems … we won't intervene further.

#### Subtheme 1.3: the fear of medical disputes and liability uncertainty

Frontline professionals reported that family members of cancer survivors often criticize and find fault with their attitude and efficiency, questioning their professionalism in providing psychosocial care. Moreover, we have identified participants' worries that misunderstandings during communication with family members could lead to disputes, prompting them to withhold certain information.

N7 In daily work, we often encounter some picky family members, and it feels really frustrating. I've already put so much effort into caring for the patient, so why do the family members criticizing me, I would rather avoid these risks.

N3 We do provide psychosocial advice, but if people perceive it as overstepping, we refrain from offering further input. We avoid causing trouble—if our advice is misunderstood or leads to complaints, the responsibility ultimately falls on us.

### Theme 2: emotional and social struggles faced by survivors and their families that hinder the employment of psychosocial care

#### Subtheme 2.1: communication difficulties

A number of participants identified communication difficulties as a major obstacle preventing survivors and their families from receiving psychosocial help. Nurses and social workers are expected to build strong relationships, understand survivors' and their families' needs, and provide appropriate support. However, reluctance to communicate with some survivors or family members often hinders this process. In some cases, survivors require the professionals to fulfill their requests, such as “serve me tea” or “take me for a CT scan.” These requests are beyond the clinical team's responsibilities. These interactions potentially reflected the survivor's misunderstandings of psychosocial oncology care. The communication difficulties related to misunderstandings affected the relationship building and information acquisition between professionals and survivors.

N3 From the perspective of being a patient, they feel unwilling to communicate with you.

N12 Some patients come in with a pre-existing hostile mindset. For example, they thought everyone should revolve around them, and they feel like they should be treated like a VIP. Once, there was a patient who, while we were in the hallway, said, “You nurses should serve me tea and take me to the CT room.”

N2 No matter how much we try to explain, they don't want to listen because they don't want to communicate with anyone. They just stay silent.

#### Subtheme 2.2: emotional instability, isolation, and denial of psychosocial care

We identified that most of the participants experience that survivors and their families often experience emotional instability and self-isolation when facing cancer. Some survivors show a negative attitude toward treatment and any psychosocial care, believing it to be useless and a waste of money and refusing to cooperate. Others have short tempers and resist establishing a rapport with nurses or social workers. Additionally, younger patients and their families often find it harder to accept the illness, frequently displaying sadness and denial. Although frontline professionals do their best to communicate, their efforts have limited effectiveness, highlighting the need for more specialized psychosocial care dealing with both survivors and their families in such circumstances.

S2 Some patients vent their negative emotions onto me but reject other communication and help … Some anxious patients have a low willingness to accept psychosocial care, they would like to leave here only.

N1 No matter how you try to communicate, it doesn't work. They immediately change their attitude and threaten to file a complaint … Patients' emotions are unstable, with large fluctuations, making communication quite difficult.

N6 We try to use scales to assess them, but they strongly resist this (whether patients or family members). They feel it's completely meaningless.

N7 Some patients have a bad temper and are very stubborn. They push people away right from the start. It's hard to build a relationship with them, and it even makes you want to stay far away.

#### Subtheme 2.3: the dynamic between survivors and their families

Dynamic factors, such as conflicts and caring burdens between survivors and their families, can lead to the survivor's refusal to cooperate with treatment plans. Family members may also feel helpless and anxious, struggling to care for the survivors. Specifically, our participants pointed out that family members may be overly involved out of concern for the survivor's condition, which can cause resentment from the survivors. Additionally, family relationships may be affected, as survivors might exhibit negative emotions and behaviors toward their loved ones, requiring patience and understanding from their families. These complex dynamics might serve as a factor that disrupts or affects the effectiveness of engagement of the frontline professionals in caring for survivors' psychosocial needs throughout their treatment journey.

N2 After chemotherapy, the body starts to experience various adverse reactions, so the family members become very scared and desperately push the child to eat. But the child says, “I just can't eat,” and this creates tension within the family. The child develops a rebellious attitude and refuses to talk, including with family members, nurses, and volunteers.

N14 What the mother tells the child and what the child understands (about cancer knowledge/death) are different. Then, the child feels that his mother is lying to him, and he refuses treatment, even refusing to have his blood drawn. We arranged a psychological consultation then, but it didn't help.

### Theme 3: system failures: social, structural and policy barriers

#### Subtheme 3.1: insufficient policy and institutional support for psychosocial care

The most common perceived system barriers to psychosocial care for Chinese cancer survivors occur at the institutional level. The underdeveloped psychosocial services and inadequate referral systems in mainland China fail to provide clear guidelines for nurses or social workers. As a result, those clinical staff can only rely on their personal capabilities to support survivors seeking help. Moreover, participants also stated that existing care and follow-up evaluation largely focused on the clinical aspects, such as nutritional conditions and control of side effects. In the absence of structured psychosocial care, survivors' psychological and social well-being is frequently left to self-management and family-motivated support.

N4 From what I know, their (follow-up team) only provides physical health-related information.

N3 Not only survivors' mental state, in fact, many family members also have poor psychosocial states. But there's no way to say that the hospital can provide specific support.

N6 Survivors have to deal with the psychological impact of their body image on their own. They likely must process their distress by themselves, as there's no established mechanism to address these issues.

N10 Most of the time, it's up to the patients to find their own way out … What we can offer to patients now is still very limited. They have WeChat groups among themselves … so they can find someone who shares their experience or has walked the same path.

#### Subtheme 3.2: low public awareness of psychosocial care

Throughout the cancer survivorship journey, the participants observed that many cancer survivors and their families have low awareness of psychosocial care, which hinders their help-seeking behavior. Specifically, some survivors view psychosocial distress as an inevitable or “the only way” of cancer experience. Additionally, the stigmatization surrounding psychosocial care might lead to worries about discrimination or labeling, such as “people having problems” and “depression,” contributing to reluctance in seeking care. Moreover, some family members also believe that withholding a cancer diagnosis is beneficial for survivors' treatment and reduces the burden on professionals. This reflects a Chinese cultural norm that emphasized “not causing trouble to others”. In general, low public awareness, combined with social and cultural influences, continues to limit cancer survivors ' access to psychosocial oncology care.

S2 In Asian culture, the traditional value of “not causing trouble for others” makes it difficult for survivors to share their fears and pain with others … I still remember a case where the survivor hid her condition from their children. As a result, she can only seek treatment secretly and vent their emotions (crying) in the hospital without their family's support.

N3 I think most Chinese people, actually, have some resistance to psychosocial interventions. They feel it's normal (struggles) or that having thoughts is their personal issue and they shouldn't seek help.

S1 The survivors always feel, “Why do I need someone's help? I don't need it.” They thought seeking psychosocial help was harmful to self-esteem and fear of being stigmatized by society.

#### Subtheme 3.3: financial burden and insurance gaps in psychosocial care

Many nurses and social workers reported that high costs and insurance gaps significantly limit access to psychosocial care in China. The financial costs of hospital operations include the need for profitability and a high ward turnover rate. Challenging to invest long-term and additional resources in employing psychosocial care. Although some hospitals offer counseling services, limited people have access to these services due to the high turnover rate and financial considerations. Basic health insurance in mainland China does not cover psychosocial services, given the high costs of medical cancer treatment, cancer survivors and their families prioritize physical health care over psychosocial care. While charitable funds and financial aid programs exist, strict eligibility requirements make them inaccessible to many. Overall, economic barriers represent key systemic obstacles preventing cancer survivors from accessing the psychosocial care they need.

S3 The difficulties are regarded as economic factors—we lack practical support for psychosocial care, particularly for the financial strain. Many people actually give up treatment because their families can't afford it.

N12 Psychosocial consultations cost money … a specialist nurse in psycho-oncology … is charged 500 yuan per hour … and it's reportedly not covered by national insurance.

N10 The hospital bed turnover rate is high, we can do nothing for psychosocial care—more patients are waiting for those beds.

N14 We don't have such resources or connections (psychosocial care). The hospital is controlling costs, and national insurance is cutting our funding … everything is tightly restricted.

N8 Psychosocial care is a loss-making service. The hospital has to subsidize this work because the income can't cover medical staff salaries or other expenses. There's no way to sustain this long-term.

#### Subtheme 3.4: geographical barriers to accessing care

According to participants' frontline experiences, geographical inequalities in medical resources affect cancer survivors' access to psychosocial care. While major cities in mainland China offer state-of-the-art cancer treatment, high patient turnover, overwhelming demands, and an inadequate referral and follow-up system make it challenging to track the survivors once discharged from the hospital. As a result, the geographical barriers hinder the equitable and accessible provision of psychosocial services in mainland China.

S3 These survivors came from all over the country … After discharge, their rehabilitation is done at home … Follow-ups are usually scheduled every six months to a year, it all depends on the survivors' self-discipline. It's a mindset—they feel that once they're “cured,” they no longer need social workers' assistance, let alone undertake long, arduous trips.

S2 Actually, I notice the unequal distribution of psychosocial care resources in regions and healthcare institutions, particularly in rural areas. These barriers limited survivors' timely and effective access to these supports.

## Discussion

**Fig. 1:**
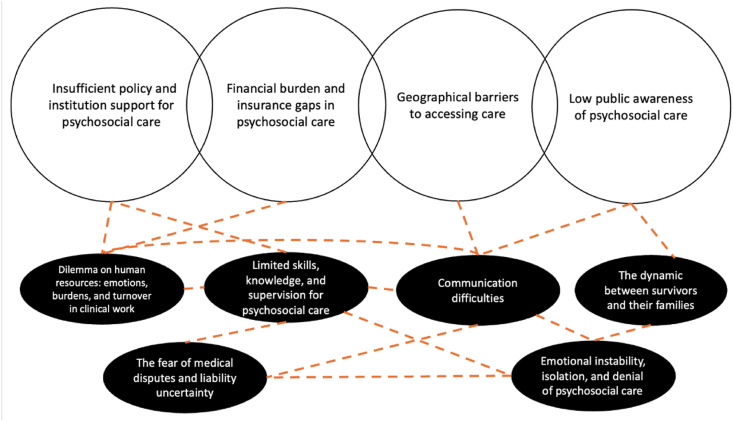
Visualization of the interrelationship between subthemes.

The findings revealed three key themes and subthemes that shed light on the barriers to providing psychosocial oncology care from the frontliners' perspective. First, overburdened clinical work and inadequate training in psychosocial care lead to emotional stress and high turnover among frontline professionals. Second, survivors and their families presented challenges that complicate the delivery of psychosocial care. Third, system failures appear to underlie the first two themes, offering more profound insight into the structural barriers facing frontline professionals and contributing to the persistent unmet needs of Chinese cancer survivors. These findings illustrate how individual-level barriers are embedded within broader social and structural limitations, offering a comprehensive understanding of multifaceted barriers that hinder the provision of psychosocial care to Chinese cancer survivors. This discussion further examined the linkage and interrelations between themes and directed to the insights of this study. Fig. 1 utilizes the Code Relations Browser of MAXQDA^39^ to visualize the interrelationship between subthemes. This figure demonstrates the linkage between structural barriers and professionals' perceptions of challenges in providing psychosocial oncology care. The themes in the top row illustrate primary structural challenges that contribute to individual challenges depicted in the lower row of themes. The themes in the lower row highlight the practical difficulties experienced by professionals. These, in turn, result in limited access to psychosocial care for cancer survivors. The dashed lines represent the interrelations among these themes, suggesting a multidimensional, mutually reinforcing set of barriers that hindered the provision of Chinese psychosocial oncology.

Chinese frontline professionals in psychosocial oncology care reported experiencing complex barriers in their daily work, spanning clinical, interpersonal, and structural levels. These findings align with previous research on the challenges for Chinese oncology nurses in providing hospice care.^32^^,^^40^ Despite efforts to develop interdisciplinary oncology care, its implementation remains hindered by structured barriers and a lack of common understanding.^32^ We highlighted the importance of distinguishing between psychosocial care and hospice care in terms of goals, setting, and duration. Psychosocial care focuses on ongoing support for the psychological and social aspects throughout cancer treatment and recovery.^41^ In contrast, hospice care provides curative treatment, symptom management, and spiritual support for advanced cancer patients in the final months or weeks of life.^40^ Psychosocial oncology care has broader coverage and is significantly underdeveloped compared to hospice care in mainland China.^19^^,^^40^ The cultural and systemic challenges further complicate frontline professionals' delivery of psychosocial care, including limited policy and funding support and inadequate training.^16^^,^^19^ Without this support, cancer survivors' psychosocial distress continues to be underestimated in mainland China.^19^ Importantly, cancer survivorship entails not only medical rehabilitation but also the reconfiguration of the psychosocial well-being of survivors.^1^^,^^4^^,^^11^^,^^18^ When psychosocial needs go unmet, cancer survivors and their families may feel ill-equipped and disempowered to cope with the challenges of cancer survivorship.^4^^,^^18^ This sense of helplessness can also hinder relationship building with care providers and heighten concerns about medical disputes. This evidence highlights a troubling paradox: while a rapport relationship is essential for psychosocial care, the time and resources available to frontline professionals and survivors are severely limited. As a result, professionals may prioritize survivors with readily engaged cases, while avoiding more complex, demanding cases to minimize risk and conflicts. This practice may inadvertently contribute to disparities in care and reinforce systemic inequities in psychosocial oncology.

The development of a Chinese-context-specific psychosocial oncology care framework and integrated medical treatment and psychosocial oncology care is not a direct process. Our findings highlight that frontline professionals face substantial barriers to providing psychosocial oncology care, which are interrelated with structural factors. Although we identified that “Psychosocial Liaison” positions (e.g., participants N9, N11, N12, N13, N14, N16) were established in some oncology hospitals in mainland China, broader impacts of these efforts remain limited by resources and geographical inequalities.^19^^,^^32^ Despite professionals' commitment to provide psychosocial oncology care, the high healthcare costs, high demands of medical treatment, and the insurance gaps for psychosocial care make survivors and hospitals flinch to offer/accept these services.

In addition, the difficulties encountered in helping cancer survivors and family members understand psychosocial oncology care are largely relevant to the Chinese cultural context. The Chinese cultural values, such as endurance in the face of hardship (“tolerance for struggle”) and avoidance of burdening others (“not causing trouble to others”), are generally challenged by survivors seeking psychosocial help.^16^^,^^19^ These cultural dynamics are also reflected in communication difficulties and resistance to care among frontline professionals and survivors. Moreover, our findings also indicated the complex dynamic between Chinese cancer survivors and their families throughout the cancer survivorship journey, which aligned with Wang and Li.^42^ Specifically, the absence of formal psychosocial care, Chinese family caregivers undertake a wide range of duties for caring for cancer survivors, including psychosocial support for survivors. Their fatigue and stress may, in turn, affect how survivors cope with cancer.^42^ To address these challenges, Chinese psychosocial oncology care should incorporate psychoeducation and community-based support for family caregivers to reduce their burdens in caregiving.^42^ Future research should also explore survivors' and families’ understanding and perceptions of psychosocial care. Overall, these findings call for actions to enrich the public and institutional awareness of psychosocial oncology care and assist and empower frontline professionals in delivering psychosocial care.^32^

Despite numerous barriers, frontline professionals in mainland China continue to provide psychosocial oncology care by utilizing their own skills, knowledge, and available resources. Their commitment reflects professionals' deep awareness and belief in the important role of psychosocial care in supporting cancer survivorship. As Tieu^43^ suggested, cancer survivorship should be viewed as a life course process, indicating healthcare systems should be innovatively designed to meet the long-term and individualized health-related challenges. This perspective suggests that the society, healthcare systems, and policies should collaborate to solve the barriers of frontline professionals in China and to meet the increasingly complex psychosocial needs of Chinese cancer survivors. These results emphasized the need to develop a Chinese-context-specific psychosocial oncology care framework.

### Implications for clinical practice and research

Our findings offer significant implications for future policy, practices, and research in developing a Chinese-context-specific psychosocial oncology framework.

First, this study highlighted the gaps and needs for examining the underdeveloped psychosocial oncology within the Chinese context. The structural and cultural barriers to providing psychosocial oncology care still exist, compared with previous studies in the past decade,^16^^,^^20^ and correspond to the findings of this study. We underscore the importance of psychosocial oncology within the Chinese context; it should be collaborated with research in promoting the intervention, policy, and clinical and social education that enrich the public awareness of psychosocial oncology care.^43^

As Pirl et al.^7^ argued, it is critical to establish or refer to an institutional framework for planning services and allocating resources, aims, and scope of psychosocial oncology. While small numbers of social workers contribute to this work, current practices indicate that nurses are dominant forces in screening, intervening, and referring survivors with psychosocial needs. Given the heavy burdens of Chinese nurses, these structures might be unhealthy and unsustainable, which is reflected in the emotional distress, burden, and turnover of the participants. We align with Tang et al.^19^ that the interdisciplinary cancer treatment team might be a feasible solution. The emerging consultation-liaison psychiatric service in mainland China demonstrated the possibility of involving different specializations, such as clinical psychologists, during cancer treatment.^25^ Additionally, oncology social workers as a crucial supplement, could serve as professionals who solve the diverse and complex psychosocial needs of cancer survivors.^19^ Some social worker participants indicated that the current framework of oncology social work in mainland China is not institutionally authorized and that they are working under civilian foundations rather than a national healthcare institution. We highlighted a psychosocial oncology framework balancing resources, aims, and the scope of the service, and oncology social work should be included as a necessary part of the current Chinese cancer treatment. Moreover, we suggested that normative psychosocial oncology services potentially reduce survivors' stigmatization and enrich public awareness.^32^ In this view, policies should extend insurance coverage on psychosocial aspects in health care providers at the commercial, provincial, and national levels, minimizing the financial hardship for cancer survivors in China.^44^ Moreover, to reduce the burden of frontline professionals, the function of community health service centres needs to be reformed to address the high volume of psychosocial needs of discharged cancer survivors.^26^

This study called for standardized psychosocial oncology in nursing education to allow nursing students to enrich their skills and knowledge of psychosocial oncology in coping with the current dilemma. For example, the continued training for frontline professionals can utilize the existing handbook of psychosocial oncology, such as “Chinese Clinical Practice Guidelines for Psycho-Oncology (2020)”. The innovative methods, including online peer support groups, narrative approaches, and photovoice approaches, should be shared with professionals to facilitate the engagement or intervention for cancer survivors.^4^^,^^18^^,^^45^

### Strengths and limitations

This study strengthened the gap in the barriers to Chinese psychosocial oncology care from the perspective of frontline professionals. The first-hand experience of frontline professionals contributes to demonstrating their encounters with challenges, which directly informs policymakers to review and reform the dilemma in Chinese psychosocial oncology care.

It is important to acknowledge several limitations of this study. First, the small sample size with a wide range of spatial distribution and specialization may lead to findings that do not reflect the generalizability and comprehensive frontline barriers of psychosocial oncology care. Specifically, our participants were dominantly recruited from developed cities (i.e., Guangzhou, Shanghai, and Beijing) and top-tier public hospitals (Sanjia Hospital: Class III Grade A Hospital, the highest tier in China's healthcare system) in mainland China, which might not present the circumstances in other regions and lower levels of hospitals. Second, the diversity of specialization of these frontline professionals indicates they might have a divided perspective on psychosocial oncology care. To solve these problems, the authors clearly defined psychosocial oncology care concepts for each participant. Third, participants' vast differences in working experiences (i.e., 1–20 years) might affect the comprehensiveness of data interpretation. We only recruited four professionals with less than 3 years of experience to control the number of less-experienced professionals to balance potential concerns about data interpretation. Finally, we acknowledged that the first author's perspective may have been influenced by personal experience as a cancer survivor. These limitations suggest that future research should unearth the nuances and differences between frontline professionals in distinct specializations, regions, and experiences. To generate a more comprehensive understanding of barriers to the implementation of psychosocial oncology care.

## Conclusions

This study employed a thematic analysis approach to explore the frontline professionals' barriers to providing psychosocial oncology care within the Chinese context. The findings revealed three key challenges: (1) overburdened clinical work and inadequate training, (2) emotional and social struggles faced by survivors and their families that hinder the employment of psychosocial care, and (3) system failures in insufficient institutional support, low public awareness, financial burdens and gaps, and geographical inequalities. A central paradox emerged between frontline professionals' desire for more comprehensive psychosocial care and the interrelated systemic limitations imposed by current institutional and societal structures. These findings emphasized the need for further study and the development of a tailored psychosocial oncology framework within the Chinese context. Specifically, the Chinese government should play a leading role in integrating psychosocial oncology care into broader healthcare systems to ensure institutional and financial support in implementation. We underscore the need for a context-specific framework integrating interdisciplinary teams, including oncology social workers and clinical psychologists, to better address survivors’ complex needs. Finally, standardized training and innovative methods are a key step in normative psychosocial oncology services, which potentially facilitates public and institutional awareness for engaging psychosocial care. Overall, the frontliners' perspectives call for multiple-level actions within Chinese healthcare institutions to deliver effective psychosocial oncology care.

## CRediT authorship contribution statement

**Ziqi Peng**: Conceptualization, Methodology, Software, Validation, Investigation, Formal Analysis, Resources, Data Curation, Writing – Original Draft, Writing – Review & Editing, Visualization, Project Administration. **Xiaohui Su:** Validation, Investigation, Formal Analysis, Resources, Data Curation, Writing – Original Draft, Writing – Review & Editing. **Suet Lin Hung:** Supervision. All authors have read and approved the final manuscript.

## Ethics statement

The study obtained ethical approval by the Research Ethics Committee (REC) of the Faculty of Arts and Social Sciences at Hong Kong Baptist University (Ref No. FASS-SOWK-24-25-001) and was conducted in accordance with the 1964 Helsinki Declaration and its later amendments or comparable ethical standards. All participants provided written informed consent.

## Data availability statement

The data that support the findings of this study are available upon reasonable request from the corresponding author, ZP. The data are not publicly available due to ethical restrictions.

## Declaration of generative AI and AI-assisted technologies in the writing process

No AI tools/services were used during the preparation of this work.

## Funding

This study received no external funding.

## Declaration of competing interest

The authors declare no conflict of interest.
